# Understanding the Effects of Antipsychotics on Appetite Control

**DOI:** 10.3389/fnut.2021.815456

**Published:** 2022-01-03

**Authors:** Sayani Mukherjee, Silje Skrede, Edward Milbank, Ramaroson Andriantsitohaina, Miguel López, Johan Fernø

**Affiliations:** ^1^Hormone Laboratory, Haukeland University Hospital, Bergen, Norway; ^2^Department of Clinical Science, University of Bergen, Bergen, Norway; ^3^Section of Clinical Pharmacology, Department of Medical Biochemistry and Pharmacology, Haukeland University Hospital, Bergen, Norway; ^4^NeurObesity Group, Department of Physiology, Center for Research in Molecular Medicine and Chronic Diseases, University of Santiago de Compostela-Instituto de Investigación Sanitaria, Santiago de Compostela, Spain; ^5^CIBER Fisiopatología de la Obesidad y Nutrición, Centro de Investigación Biomédica en Red de la Fisiopatología de la Obesidad y Nutrición, Madrid, Spain; ^6^SOPAM, U1063, INSERM, University of Angers, SFR ICAT, Bat IRIS-IBS, Angers, France

**Keywords:** hypothalamus, appetite control, mechanisms, hyperphagia, antipsychotics

## Abstract

Antipsychotic drugs (APDs) represent a cornerstone in the treatment of schizophrenia and other psychoses. The effectiveness of the first generation (typical) APDs are hampered by so-called extrapyramidal side effects, and they have gradually been replaced by second (atypical) and third-generation APDs, with less extrapyramidal side effects and, in some cases, improved efficacy. However, the use of many of the current APDs has been limited due to their propensity to stimulate appetite, weight gain, and increased risk for developing type 2 diabetes and cardiovascular disease in this patient group. The mechanisms behind the appetite-stimulating effects of the various APDs are not fully elucidated, partly because their diverse receptor binding profiles may affect different downstream pathways. It is critical to identify the molecular mechanisms underlying drug-induced hyperphagia, both because this may lead to the development of new APDs, with lower appetite-stimulating effects but also because such insight may provide new knowledge about appetite regulation in general. Hence, in this review, we discuss the receptor binding profile of various APDs in relation to the potential mechanisms by which they affect appetite.

## Introduction

Schizophrenia, a chronic psychotic illness with a lifetime prevalence ~0.7%, is associated with a long-term reduced quality of life for affected individuals and constitutes a major socio-economic challenge worldwide (1). For patients suffering from schizophrenia, the life expectancy declines by 15–20 years in comparison to unaffected individuals (2). Somatic conditions such as cardiovascular disorders (CVD) have been estimated to account for as much as 60% of the mortality gap (3). In fact, while CVD accounts for 33% of deaths in the general population, the corresponding figure for persons diagnosed with schizophrenia is 75% (4).

Pharmacological treatment with antipsychotic agents is available for the so-called positive (psychotic) symptoms of schizophrenia, such as hallucinations and delusions. The core property of all antipsychotic agents is dopamine 2 (D2) receptor antagonism (or partial agonism) in the central nervous system (CNS) (5). First-generation antipsychotics (FGA), introduced in the early 1950's, can cause serious extrapyramidal adverse effects such as dystonias, parkinsonism, and tardive dyskinesias, the prevalence of which is correlated with the degree of dopamine receptor blockade (6). The so-called second-generation antipsychotics (SGA), introduced from 1993 onwards, are associated with a lower risk of motor dysfunction (7). However, some of these drugs, in particular clozapine and olanzapine, have increased propensity to induce adverse metabolic effects such as pronounced weight gain, dyslipidemia, and diabetes (8, 9). Current evidence indicates that the observed weight gain is largely due to hyperphagic effects of antipsychotics, with a lack of satiety observed in patients and rodent models (10–14). Metabolic adverse effects likely contribute to excess mortality from cardiovascular disease (CVD) in patients, constituting a pressing patient safety issue in psychiatry (4). Interestingly, despite the association of olanzapine and clozapine with a more pronounced risk of weight gain and metabolic adverse effects, they remain widely used because many patients respond well to these agents with regard to symptomatic relief and tolerability (6, 8).

Most antipsychotics have an affinity for a broad range of neurotransmitter receptors in the CNS in addition to the dopamine receptor, some of which have been associated with metabolic side effects. In particular, the risk of weight gain and glucose intolerance has been linked to an affinity to CNS histaminergic (H1), serotonergic (5HT1, 5HT2a, and 5HT2c), and muscarinic M1 and M3 receptors (5, 13, 15). A growing body of publications attempts to unveil the molecular effects of antipsychotics on appetite regulation. The focus of this review is to present an overview of how the receptor binding profiles of various APDs may explain their orexigenic effects and to elucidate the intracellular pathways involved in the regulation of appetite.

## The Hypothalamus as Master Regulator of Appetite

**Figure 1:**
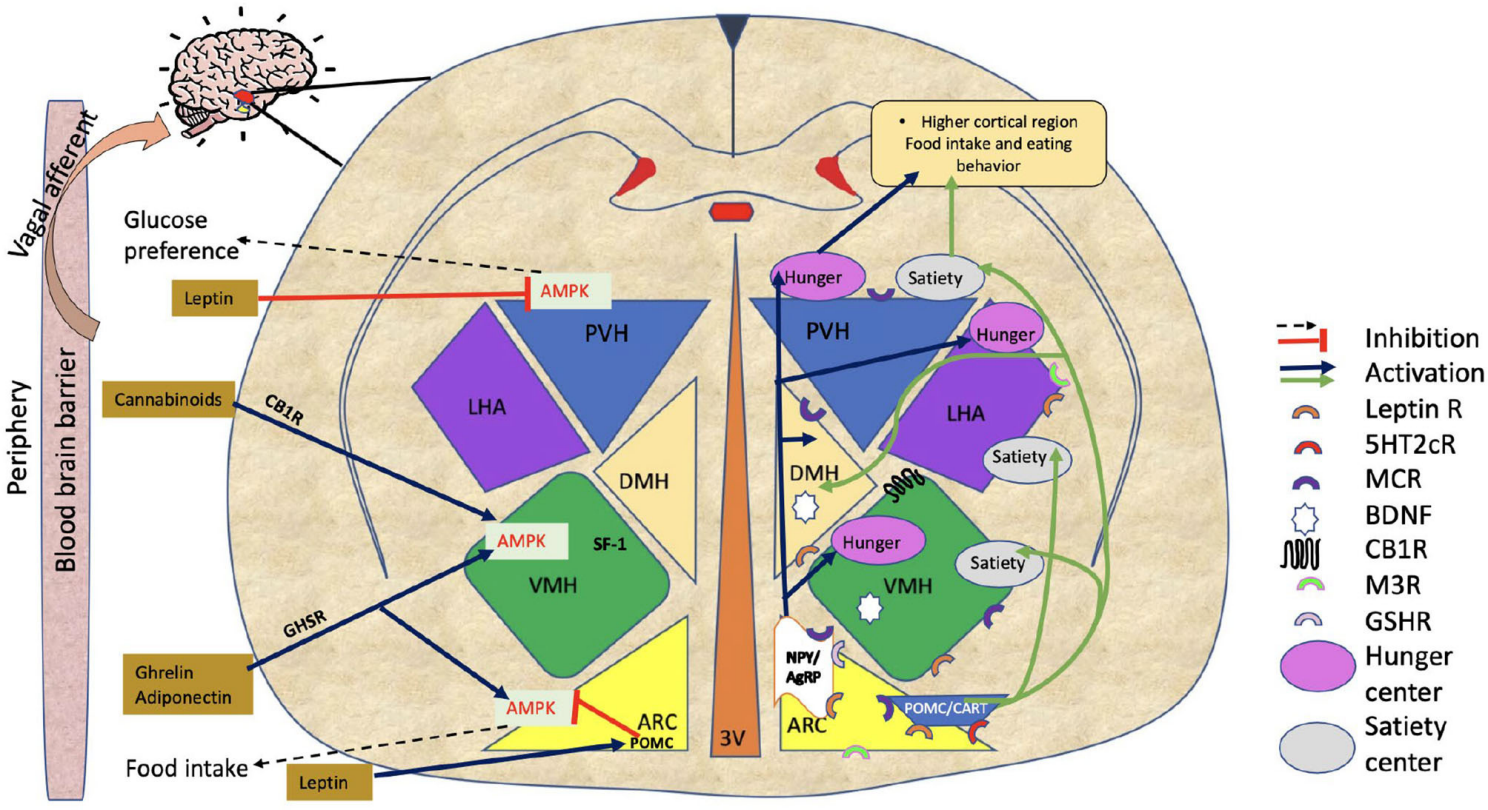
Hypothalamic regulation of food intake. The figure shows hypothalamic regulation of food intake involving various hypothalamus regions (ARC, VMH, LHA, PVH, and DMH). The NPY/AgRP pathway increases food intake, while the POMC/CART pathway triggers satiety. Adiponectin/ ghrelin, and cannabinoids all demonstrate food intake regulation by AMPK activation in the ARC or in VMH. Leptin reduces food intake by directly inhibiting AMPK in PVH or indirectly *via* POMC neurons in ARC. Black dotted arrows and red lines with blunted ends are showing inhibitory signals. Stimulatory signals are represented by blue and green arrows. On the figure, half circles in different colors show the locations of various receptors through which APDs work. ARC, Arcuate nucleus; VMH, Ventromedial hypothalamus; LHA, Lateral hypothalamus area; PVH, Paraventricular nucleus of hypothalamus; DMH, Dorsomedial hypothalamus; NPY/AgRP, Neuropeptide Y/ agouti-related peptide; POMC, Proopiomelanocortin; CART, Cocaine and amphetamine-regulated transcript; AMPK, AMP-activated protein kinase; M3R, Muscarinic 3 receptor; BDNF, Brain-derived neurotrophic factor; GSH-R, Ghrelin receptor; MCR, Melanocortin receptor; CB1R, Cannabinoid receptor; 5HT2cR, 5-hydroxytryptamine 2c receptor (serotonin receptor); Leptin R, Leptin receptor; APDs Antipsychotics.

In order to understand the relationship between APD receptor binding profile and their propensity to modulate appetite-regulating mechanisms, it is important to understand the CNS anatomy where these interactions take place. An individual's body weight and food intake are primarily controlled by the hypothalamus (Figure 1) (16, 17). The hypothalamus consists of anatomically distinct groups of neurons known as nuclei, including the arcuate nucleus (ARC), ventromedial hypothalamus (VMH), paraventricular nucleus (PVH), and lateral hypothalamus area (LHA) (18). Integrating a wide array of afferent signals, the hypothalamus regulates energy intake and expenditure through signaling mediated by neuromodulators. These include (i) the anorexigenic neuropeptides proopiomelanocortin (POMC), a polypeptide precursor to the endogenous melanocortin receptor agonists (α- and β- melanocyte-stimulating hormones) (19) and cocaine and amphetamine-regulated transcript (CART) (16, 20–23) and (ii) the orexigenic neuropeptide Y (NPY) and agouti-related peptide (AgRP) that acts as an endogenous antagonist and inverse agonist of melanocortin receptors (MC4R) (24). The ARC is considered one of the most important hypothalamic regulators of feeding and contains both anorexigenic and orexigenic neurons. ARC lies adjacent to the third ventricle and median eminence that plays a significant role in metabolic sensing as it contains partially permeable blood-brain barriers, and neurons in this region connect the periphery with the central nervous system.

The PVH is shown to be involved in neuroendocrine activity, as it produces neuropeptides such as oxytocin (OXT), thyrotropin-releasing hormone (TRH), and corticotropin-releasing hormone. These hormones have traditionally been considered secondary to those controlling food intake and energy expenditure circuitry. Neurons in these circuits respond to energy shortages in two ways. First, they modify the flow of neuroendocrine hormones. Second, they alter the role of preganglionic neurons in the brainstem and spinal cord. Their connections allow them to control energy balance by affecting peripheral tissues, such as the pancreas, liver, spleen, white adipose tissue (WAT), and brown adipose tissue (BAT) (25). Additionally, like ARC, PVH also regulates energy balance by POMC and NPY/AgRP neurons. In the PVH, AgRP neurons stimulate feeding by inhibiting single-minded 1 (SIM1)-expressing target neurons, and activation of POMC exhibits inhibition in food intake (26). It has been reported that SIM1 is also essential for the developmental growth of neurons in the PVH, thereby playing a role in energy balance (27).

The VMH is also significant in regulating glucose metabolism and appetite control (28). VMH neurons express leptin receptors (LEPRs) and estrogen receptors (ERs) (29). According to reports (30), leptin causes the steroidogenic factor-1 (SF1)-positive neurons in the VMH to depolarize and fire more rapidly. Using mice lacking LEPRs on SF1-positive neurons, it has been shown that leptin acts at this site to reduce body weight. These findings emphasize the role of VMH neurons in controlling food intake and diet-induced obesity.

## The Mesolimbic Pathway in the Regulation of Food Intake

**Figure 2:**
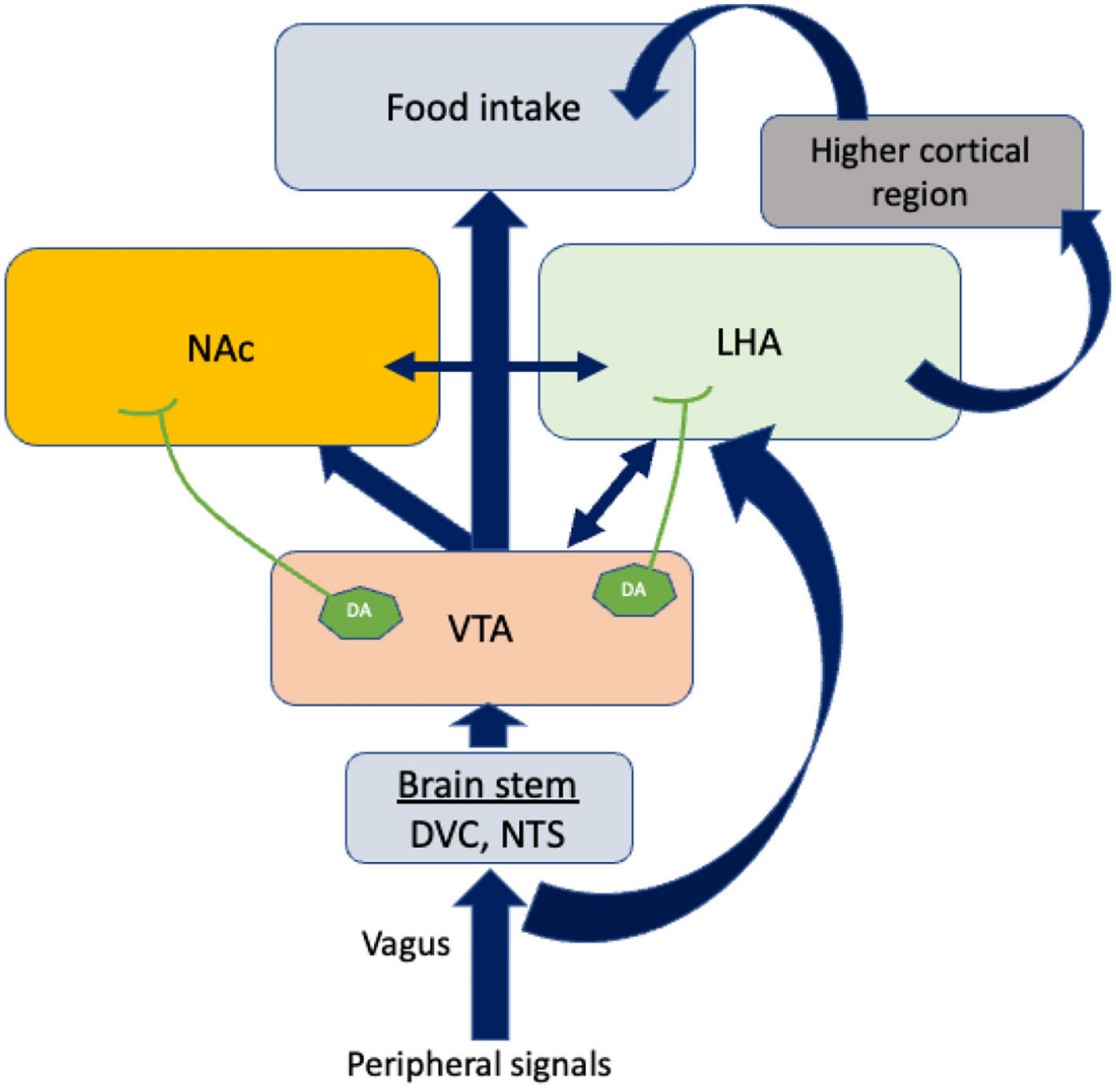
The regulation of food intake by other brain areas. Vagal afferents bring peripheral signals into the VTA by crossing the brain stem. Dopamine neurons (DA) in the VTA project axons to LHA and NAc. In response to the dopamine neurons projected by VTA, LHA connects higher cortical regions to control food intake. The blue arrows indicate the connections between the different brain areas. The double-sided arrows indicate bidirectional connections. VTA, Ventral tegmental area; NAc, Nucleus accumbens; LHA, Lateral hypothalamus area; DA, Dopamine neurons; DVC, Dorso vagal complex.

Feeding behavior is also influenced by the mesolimbic pathway (Figure 2). The ventral tegmental area (VTA), positioned in the midbrain, and the nucleus accumbens (NAc), placed in the forebrain, are the two most important structures of this system. The VTA contains dopamine neurons that connect to the NAc. It has been reported that glutamatergic and cholinergic input stimulates dopaminergic neurons, while γ-aminobutyric acid GABAergic input inhibits them (31). Dopamine release at the pre-synaptic level is likely to be responsible for “rewarding” (32). In addition to dopaminergic transmission, cholinergic interneurons deeply penetrate NAc neuronal cells and inhibit dopamine-induced food intake (33).

## Brain Stem in the Regulation of Appetite

The caudal brain stem has been shown to play a predominant role in eating processes since it contains all the motoneuron cells involved in different patterns of eating behaviors such as swallowing, suckling, chewing, and licking. Dorsal vagal complex (DVC) premotor neurons are responsible for generating these ingestive patterns. There are three areas in the DVC: the nucleus tractus solitarii (NTS), the area postema, and the dorsal motor nucleus of the vagus nerve. NTS subnuclei are of particular interest because they are the sites where peripheral signals are integrated with motor neurons (Figure 2). Therefore, neurons in the NTS receive dense mechanoreceptor input from the upper gastrointestinal tract (34). Thus, the caudal brain stem comprises some of the normal machineries of hunger and satiation previously believed to be confined to the hypothalamus and the forebrain (35). Further, recent research into neuropeptides, specifically their role in eating behavior, suggests that the caudal brain stem possesses leptin and insulin receptors, glucose-sensing pathways, and neuropeptide mediators that contribute to energy metabolism (1, 36, 37).

## Regulation of Appetite by APDs Through Neurotransmitter Systems

### The Serotonergic System

Serotonin is a neurotransmitter that regulates food intake and energy expenditure. The first link between serotonin and appetite was described more than 35 years ago (38). As part of the body's homeostatic circuitry, serotonin regulates metabolic signals that communicate energy status and suppress appetite when the body meets its energy requirements. POMC and NPY neurons have been reported to contain serotonin receptors that regulate food intake (39, 40). It has been shown that 5HT2c receptors and serotonin work together to stimulate POMC cleavage, and the inhibition of NPY and AgRP by serotonin at the 5HT1b receptor hinders GABAergic transmission, thereby inhibiting α-MSH and resulting in satiety and thermogenesis.

**Figure 3:**
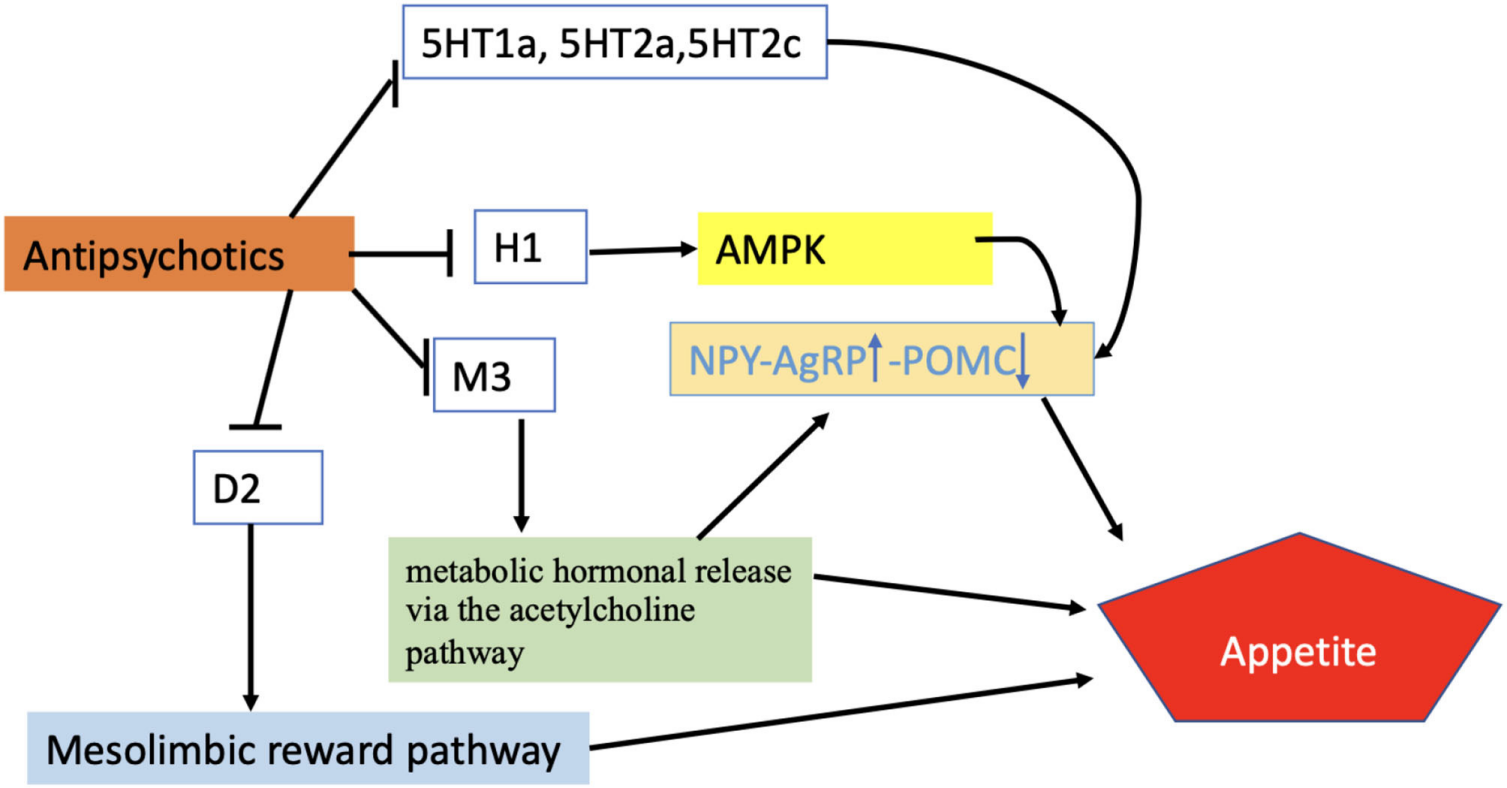
Regulation of appetite by APDs. According to the proposed scheme, APDs enhance appetite through different receptor systems activating AMPK-NPY system, metabolic hormonal release *via* Ach pathway, or mesolimbic pathway. Blunt-ended black lines indicate inhibitions. Black arrows indicate stimulations. The upward arrow represents the upregulation of the NPY/AgRP signals while the downward arrow indicates the downward regulation of POMC. H1, Histamine receptor; M3, Muscarinic receptor; D2, Dopamine receptor; 5HT1a,2a,2c, Serotonin receptors; AMPK, AMP-activated protein kinase; NPY/AgRP, Neuropeptide Y/ agouti-related peptide; POMC, Proopiomelanocortin.

There are several lines of evidence pointing toward a role for 5HT2c and possibly 5HT1/2a receptors in APD-induced hyperphagia and weight gain (5, 41–43) (Table 1, Figure 3). It is well-known that olanzapine and clozapine show antagonism and have a high affinity for the serotonin 5HT2a and 2c receptors. Additionally, it has been shown that clozapine affects glucose homeostasis by blocking 5HT2a receptors (44). Loss of 5HT2c receptors has been shown to alter feeding behavior and to trigger obesity in mice, and to alter feeding behavior (45). The importance of the 5HT2c receptors in relation to APDs was recently demonstrated in a rodent model, where olanzapine-induced hyperphagia and weight gain are diminished in mice lacking this receptor. Also, treatment with the 5HT2c receptor-specific agonist lorcaserin suppressed hyperphagia and weight gain induced by olanzapine, suggesting pharmacological treatment with this drug as a valid strategy to counteract APD-induced weight gain (46).

**Table 1:** APDs and relationship with appetite dysregulation and obesity.

**Antipsychotics (SGAs)**	**Receptors**	**Activity**
•Olanzapine (antagonism) •Clozapine (antagonism) •Quetiapine (antagonism) •Risperidone (antagonism) •Ziprasidone and Aripiprazole (5HT1a agonism, 5HT2a, and 5HT2c antagonism)	5HT1a, 5HT2a, and 5HT2c	Obesity and dysregulated food intake (observed mainly in olanzapine, clozapine, risperidone, and quetiapine treatment) (41–46).
•Olanzapine (antagonism) •Clozapine (antagonism) •Quetipine (antagonism) •Risperidone (antagonism) •Ziprasidone (antagonism)	H1 /H3	Obesity and dysregulated food intake (observed mainly in olanzapine, clozapine, and quetiapine treatment) (56–70).
•Olanzapine (antagonism) •Clozapine (antagonism) •Quetipine (antagonism) •Risperidone (mild antagonism) •Ziprasidone (antagonism with low affinity) •Aripiprazole (partial D2 agonism)	D2	Obesity and dysregulated food intake (observed mainly in olanzapine and clozapine treatment) (71–74).
•Olanzapine (antagonism) •Clozapine (antagonism)	M3	Obesity, dysregulated food intake, and peripheral effects (77–82).

*Different APDs are shown in the table in connection with 5HT1a, 5HT2a, 5HT2c, H1/H3, D2, and M3 receptor systems in the regulation of appetite and obesity. Both clozapine and olanzapine demonstrate a strong connection between food intake and weight gain*.

*5HT1a,2a,2c, Serotonin receptors; H1/H3, Histamine receptor; M3, Muscarinic receptor; D2, Dopamine receptor; SGAs, Second-generation antipsychotics*.

Quetiapine is another antipsychotic drug with 5HT receptor antagonistic properties that is linked to a significant risk of obesity and hyperphagia (47). The receptor affinity of quetiapine is high for serotonergic 5HT2a-receptors, moderate for dopamine D2-receptors, and low for 5HT2c compared to D2-receptors (48). In a recent case study, it was reported that this receptor binding profile leads to disturbances in sleep, and that quetiapine may cause sleep-related eating disorders. However, the precise mechanism of how quetiapine facilitated sleep-related food intake remains unclear (49).

Risperidone also possesses antagonism of 5HT2 receptors as well as milder dopamine D2 receptor antagonism (50). Kursungoz et al. (51) have found that risperidone affects the expression and plasma concentrations of appetite-regulating hypothalamic peptides by inhibiting 5HT2c receptors. This conclusion is supported by a recent study by Wan and colleagues (52), demonstrating that the 5HT2c receptor-NPY pathways are involved in the stimulatory effects of risperidone on appetite and weight gain in rodents.

Ziprasidone and aripiprazole are two SGAs that are associated with little or no weight gain (53). Ziprasidone acts as an agonist at 5HT1a receptors and as an antagonist at 5HT2a, 5HT2c, and 5HT1b/1d receptors, while aripiprazole has partial agonism at 5HT1a receptors and antagonism at 5HT2 receptors. It has been reported that the coadministration of olanzapine with ziprasidone or aripiprazole does not induce food intake (54), possibly due to the partial agonism effect on 5HT1a receptors that they have in common, but other mechanisms may also be involved.

### The Histaminergic System

Some of the orexigenic effects of atypical APDs are believed to be mediated *via* their effects on histamine H1- and H3-receptors (5, 41, 55, 56) (Table 1, Figure 3). Central histaminergic activity is known to repress food intake, and it has been reported that the administration of specific H1-antagonists caused hyperphagia in both humans and rodents. Accordingly, mice lacking H1 receptors display increased food intake, altered feeding patterns, and obesity (57). In contrast, H3-receptor antagonists have the opposite effect and cause hypophagia (58).

It has been reported that olanzapine-treated rats show a reduction in H1 receptor expression levels in ARC and VMH with an increase in body weight and food intake relative to rats treated with haloperidol or aripiprazole (59). Furthermore, both olanzapine and clozapine have been reported to reduce H1 receptors levels in the VMH, with concomitant stimulation of AMP-activated protein kinase (AMPK), leading to an increase in food intake and weight gain (60). In another study, it has been reported that, by suppressing postsynaptic H1 receptors, olanzapine activates pre-synaptic H3 autoreceptors, reducing histamine synthesis and secretion, aggravating hyperphagia (61). For instance, clozapine acts on H3 auto-receptors (with moderate affinity) to block acetylcholine (ACh) and noradrenaline (NA) release, resulting in dysregulation of appetite (62). Other reports support the involvement of H1 and H3 receptors in antipsychotic-induced weight gain and food intake (63, 64). For instance, betahistine (a potent H1 and H3 agonist) has been combined with APDs (olanzapine/clozapine) and has been found to reduce APD-induced food intake and obesity both in rodents and humans through the H1R-NPY and the H1R-pAMPKα pathways (65–67).

A positron emission tomography study (PET) by Sato et al. (68) revealed high H1-receptor occupancy values in the human brain at low clinical doses of olanzapine and quetiapine, further supporting the role of the histaminergic system in controlling the appetite and obesity in humans.

In summary, direct antagonism of hypothalamic H1 receptors by SGAs can stimulate appetite involving hypothalamus-brainstem circuitry, or weight gain may be influenced by APDs partly by H3 receptors. The decrease in H1 receptor activity induced by APDs may lead to a reduction in histamine release *via* H3 receptors, thereby increasing the food intake by blocking 5HT, NA, and ACh release.

### Dopaminergic System

Dopamine antagonism (or, in the case of e.g., aripiprazole, partial agonism) is the core property of all APDs (Table 1, Figure 3). Two major dopaminergic pathways originating from the VTA (mesolimbic and mesocortical pathways) are particularly involved in hedonic feeding (69). Upon ingestion of palatable foods, dopamine is released into the ventral tegmental area of the brain, resulting in activation of the neural mechanisms connecting the VTA to the NAc by the midbrain.

Researchers have recently shown that dopamine D1 and D2 receptors (D1R and D2R) are found in POMC-positive neurons in the ARCs of rats and mice (70). Hence, these findings support that dysregulation in feeding behavior mainly relies on the dysregulation of dopamine levels as well as receptor activity that can be controlled by APDs.

It has been shown that the APD sulpiride, another D2R antagonist, increases calorie intake, whereas bromocriptine, a specific D2R agonist, counteracts this effect (71). A study by Kaur and Kulkarni (72) shows that SKF 38393 (a D1R agonist) and quinpirole (a D2R agonist) significantly reverse clozapine-induced hyperphagia. Similarly, Cho et al. (73) have shown that the availability of striatal dopamine (DA) D2/3R and body mass index are positively correlated.

Moreover, risperidone has been shown to reduce energy expenditure by antagonizing dopamine D2 receptors; thus, risperidone can also lead to weight gain by blocking D2 (74) receptors. Thus, these reports support that the dopaminergic pathway contributes to APD-induced weight gain.

Anorexia nervosa (AN) is a serious eating disorder identified with intense loss of appetite, extreme weight loss, and tremendous concern about gaining weight (75). A model for aspects of AN can be explained in activity-based anorexia (ABA), which is characterized by weight loss, hypophagia, and unusual restlessness in rodents with access to restricted food and running wheels.

It has been found that the antipsychotic amisulpride, which is also a D2/3R antagonist, prevents hypophagia and weight loss during ABA in a similar way to another D2/3R antagonist, eticlopride.

Further, amisulpride has been shown to improve weight loss tendency and hypophagia compared to olanzapine (76). Hence, selective antagonism of D2 and/or D3 receptors have been shown to reduce ABA strongly. According to a recent study on ABA by Fraga et al. (75), it has been indicated that ABA affects brown adipose tissue (BAT) and white adipose tissue (WAT) thermogenesis in significant ways. Moreover, ABA does not alter primary regulators of adipose tissue activity, such as hypothalamic AMPK or endoplasmic reticulum stress signaling, raising the question of whether dopaminergic signaling somehow affects thermogenesis that may play a role in the feeding behavior. There is still a need to determine whether this principle also applies to APD-induced weight gain.

### The Acetylcholine System

Muscarinic acetylcholine receptors are involved in appetite regulation, but few studies have looked specifically at muscarinic signaling in hypothalamic appetite regulation (77). According to Nakajima et al. (78), the M3 receptor is especially critical to the control of appetite and metabolism in the hypothalamus. M3 receptor activity involves a complex interplay between peripheral and central nervous systems, with M3 receptors controlling metabolic hormones *via* the vagus nerve linked to the hyperphagic, diabetogenic, and weight gain risks associated with APDs (Table 1, Figure 3). Correlation between muscarinic receptor affinity and weight gain risk (55), as well as correlation with the diabetogenic potential of antipsychotic medicines, has been demonstrated (79). Olanzapine and clozapine are M3 antagonists (77, 80), and both of them have been reported to increase M3R binding density in the ARC, VMH, and DVC, which results in increased weight and food intake (77). The weight gain effect of APDs through the antagonism of the M3 receptor is also supported by findings that cevimeline (an M3 agonist) reduces body weight gain caused by olanzapine treatment (81, 82).

Furthermore, direct M3 receptor antagonism in the hypothalamus and DVC by APDs (olanzapine, clozapine) has been reported to generate peripheral metabolic effects (83). Overall, these references suggest APDs' blockade of M3R in the brain may cause dysregulation of metabolic, hormonal release *via* the acetylcholine pathway, which may involve hypothalamic neurons that regulate appetite.

## Effector Molecules in APD-Induced Appetite and Weight Gain

### AMPK Pathway

Several recent studies have examined the intracellular mechanisms activated by the receptor-binding properties of APDs, as described above. There has been a great deal of attention paid to the AMPK pathway, which is pivotal in the hypothalamic energy system (Figure 1). AMPK, a cellular sensor of energy homeostasis, controls energy balance by modulating hypothalamic fatty acid metabolism (17, 23, 84–86). Increased AMPK activity is a potent orexigenic driver, whereas inhibition of AMPK, particularly in the VMH, increases brown fat thermogenesis (17, 85, 87). The physiological relevance of hypothalamic AMPK on feeding control has been elucidated by the work of Claret and colleagues that demonstrate that genetic ablation of AMPKα2 in POMC and AgRP neurons promote hyperphagic (obese) and hypophagic (lean) phenotypes (88). According to early evidence, pharmacologically or adenovirus-mediated stimulation of medial hypothalamic AMPK enhances appetite by increasing the expression of NPY and AgRP in the brain (89). In addition, hypothalamic AMPK has been shown to regulate dietary selection, first- and second-phase insulin release, lipid metabolism, and gluconeogenesis in the liver, all of which are crucial for energy balance (90–93). AMPK phosphorylation, which activates the enzyme in the hypothalamus, has been explored directly as a means of addressing central mechanisms that influence appetite and weight gain (16, 94).

A variety of receptors may regulate AMPK activity, and the role of AMPK in APD-induced hyperphagia has been examined in several studies. In a study by Kim et al. (60), it has been demonstrated that clozapine and olanzapine stimulate the hypothalamic AMPK through H1 receptor antagonistic activity. Similarly, He et al. (95) show that olanzapine activates AMPK by blocking the H1Rs in the acute stage and induces hyperphagia in female rats. To investigate the specific effect of olanzapine on hypothalamic AMPK, Skrede et al. (96) have used an adenovirus expressing a dominant-negative AMPK to inhibit AMPK-signaling in the ARC or VMH of female rats, followed by olanzapine depot injection. Interestingly, olanzapine's weight-inducing effect is attenuated by inhibition of AMPK in the ARC but not in the VMH, indicating that it is the ARC-specific AMPK activation that drives the orexigenic potential of the drug. It has been reported that olanzapine-induced AMPK-NPY orexigenic signaling could be ameliorated by betahistine co-treatment, suggesting that the H1 receptor-AMPK-NPY pathway plays a role in olanzapine-induced obesity and increase in food intake (65, 66). Furthermore, a study by Chen et al. (97) demonstrated that olanzapine increases AMPK-NPY orexigenic signaling by interfering with the interaction of H1R and ghrelin receptors (GHSR1a) in the hypothalamus of mice.

Intracerebroventricular administration of olanzapine has shown to induce hyperglycemia and activation of AMPK in the hypothalamus of mice (98) *via* α-1 adrenoreceptor and D2 receptor-AMPK axis.

### ARC Neuropeptides

Polypeptide signaling molecules are also being studied as they are crucial for hypothalamic neuron communication, food intake, and the AMPK pathway (Figure 1). Fernø et al. (99) show that sub-chronic olanzapine treatment increases NPY and AgRP in the ARC and decreases POMC, which is in agreement with the dysregulation of food intake. Palasz et al. (100) observe that the rat amygdala and hippocampal expression of NPY and POMC mRNA differ qualitatively following prolonged administration of olanzapine. Surprisingly, the POMC level in the two tested regions is significantly increased by olanzapine; however, the NPY expression does not change from control to olanzapine. As discussed above, the expression of POMC produces anorexigenic effects. Hence, this paradoxical result can only be explained by the structural and functional differences between the amygdala, hippocampus, and hypothalamus. These changes in neuropeptide expression suggest that olanzapine may also exert a pharmacological effect differently in amygdala pathways. It may be possible that antipsychotics trigger metabolic side effects through this mechanism as well.

Sezlev-Bilecen et al. (101) report that olanzapine administration in male rats decreases mRNA levels of NPY, AgRP, and POMC in the hypothalamus while CART levels are unaffected. Additionally, NPY, AgRP, and α-MSH plasma levels are decreased significantly while CART levels are also decreased. It is possible, therefore, that the inhibitory role of olanzapine on ARC neurons could be a precursor to an imbalance in neurohormone release, contributing to weight gain.

A study conducted in juvenile female rats demonstrated that risperidone (an atypical APD) failed to alter CART levels, although it increased appetite and body mass by increasing mRNA expression of NPY/AgRP in the hypothalamus (102).

We, therefore, conclude that CART, along with other ARC neuropeptides, plays a crucial role in regulating feeding, body weight, and energy metabolism. Nonetheless, the inconsistency in the CART level suggests that further investigation will be required to elucidate the exact mechanism underlying CART action by APDs.

### Endocannabinoid System in Food Intake

Throughout the last decade, research on endocannabinoids (EC) and the endocannabinoid system (ECS) has contributed to greater insight into their role in food intake (103) (Figure 1). ECS signals are mediated by CB1/CB2 receptors, as well as enzymes responsible for synthesizing and degrading the endogenous ligands attached to these receptors (104). CB1R has been shown to be highly expressed in hypothalamo-cortical and reward-related brain regions, which strongly suggests a direct role in controlling feeding (105, 106).

It has been reported that the functions of endocannabinoids are mediated by AMPK, which regulates energy balance by increasing the adenosine triphosphate/ adenosine monophosphate (ATP/AMP) ratio (107). Specifically, endocannabinoids have been shown to activate AMPK in the hypothalamus and are thus related to appetite (108). On the other hand, endocannabinoids have been shown to reduce the activity of AMPK in peripheral tissues, including adipose tissue, liver, and skeletal muscle (108, 109), leading to impaired metabolism of carbohydrates and lipids. Moreover, the reduction of AMPK can also activate lipogenesis, thereby promoting body mass and reducing energy expenditure. Consequently, CB1Rs have received the most attention because they play a crucial role in energy homeostasis and can treat obesity *via* rimonabant, a CB1R antagonist (110).

Administration of CB1R antagonists yields a reduction in food intake equally in mice with or without NPY knockout, suggesting that NPY and endocannabinoids promoted food intake independently (111–114). In other studies, it has been shown that sugar and alcohol consumption is increased in rats after administration of CB1R agonists (115, 116). On the contrary, Radziszewska et al. (117) have reported that activation of CB1R with its agonist (WIN 55, 212-2) produces anorexigenic effects. The results of these studies suggest that mostly the activation of CB receptor signaling can increase food consumption and lead to weight gain.

It has been reported that APDs alter cannabinoid receptor-binding density in the DVC of the brainstem and regulate appetite signaling and food intake. Weston-Green et al. (118) have reported that olanzapine significantly inhibits CB receptor binding in the DVC, whereas aripiprazole and haloperidol do not influence the receptor. According to these studies, olanzapine can lead to weight gain by modulating CB receptors in the DVC. According to Lazzari et al. (119), the metabolic side effects of olanzapine treatment can be neutralized by co-administration of CB1R antagonist compounds. In an investigation by Weston-Green et al. (120), it is suggested that a reduction in CB1R density during olanzapine treatment decreases cannabinoid-mediated suppression of GABA and thereby enhances GABAergic transmission to POMC neurons, reducing POMC activity and promoting weight gain.

### Brain-Derived Neurotrophic Factor

Brain-derived neurotrophic factor (BDNF) and its receptor, Tyrosine Kinase Receptor B (TyrkB) (121), are involved in the regulation of hypothalamic neural circuitry and feeding behavior and expressed in most hypothalamic nuclei, including VMH, DMH, LH, ARC, and PVH. BDNF neurons in the PVH have been shown to reduce food intake (122), although BDNF has been found to be present in the highest levels in VMH (121, 123, 124). Preclinical studies have shown that reduced BDNF levels, as well as abnormal TrkB signaling, are associated with altered food intake, excessive body weight gain, and metabolic alterations (121, 123–127). These data suggest that reduction in BDNF levels or inhibition of these neurons can lead to hyperphagia and obesity.

APDs have been shown to regulate BDNF levels, suggesting a role for this pathway also in APD-induced weight gain. In a study by Zhang et al. (128), olanzapine-treated patients with pronounced metabolic syndrome have lower serum levels of BDNF than patients with a healthier metabolic profile. In line with this, a preclinical study with haloperidol and risperidone was shown to decrease BDNF levels as well as the levels of TrkB in the brain (129). However, other findings do not support reduced BDNF associated with APD-induced orexigenic effects. In one preclinical study, both clozapine and olanzapine were shown to increase the production of BDNF in the rat brain (130), and in another study, olanzapine diminished the BDNF-lowering effect of the less orexigenic haloperidol (131). In a meta-analysis, Lin (132) show that peripheral BDNF levels are inconsistently affected by antipsychotics in patients with schizophrenia, supporting that the role of BDNF in APD-induced weight gain is complex.

Some of the inconsistent findings may be explained by gender-specific effects. E.g., in one study, BDNF levels were shown to be negatively correlated with body mass index (BMI) gain in female patients but not in male patients (133). This was supported by another study, showing that decreased BDNF serum levels were associated with weight gain in female schizophrenia patients receiving long-term antipsychotic treatment (134). In this study, in 332 patients suffering from chronic schizophrenia treated with APDs, homeostatic model assessment for insulin resistance (HOMA-IR) and BDNF are associated in a differential manner according to. Hence, it is important to consider gender when measuring BDNF levels in patients with schizophrenia and metabolic indicators (134).

## Endocrine Mechanisms in APD Induced Appetite Dysregulation

### Leptin

As a dynamic endocrine organ, the adipose tissue secretes hormones, peptides, and adipokines that regulate energy, lipid, and glucose homeostasis (135). Leptin was the first adipokine identified to facilitate a link between the adipose tissues and the hypothalamus. Increased fat mass leads to elevated secretion of leptin (136), with subsequent reduction in food intake and increase in energy expenditure (137, 138). Several areas of the hypothalamus are controlled by leptin, including the ARC, VMH, and PVH. By directly activating POMC neurons in the ARC, leptin increases the production of α-MSH. By projecting to MC4R neurons, melanocortin peptide reduces food intake and controls metabolism *via* energy storage, insulin secretion, and gastrointestinal motility. Moreover, leptin has been shown to inhibit food intake by interacting with dopaminergic neurons in the VTA (139). On the other hand, hedonic feeding suppresses satiety signals. It has been reported that mice lacking D2R were more sensitive to leptin (140–142).

Despite the anorexigenic effect of leptin, obesity is often associated with elevated leptin levels, indicating leptin resistance. When leptin resistance occurs, energy expenditure becomes unequal to intake, thereby increasing appetite and body weight. Leptin resistance may be caused by a defect in the nutritional regulation of leptin receptor gene expression, which may involve an impairment in signal transducers and activators of transcription (STAT3) signaling (143). However, even with an intact Janus kinase/signal transducers and activators of transcription (JAK2–STAT3) pathway of leptin signaling, hypothalamic neurons may exhibit leptin resistance. AMPK and acyl CoA carboxylase (ACC) are thought to be involved in leptin receptor signaling. The anorexigenic effects of leptin are shown to be dependent on inhibition of hypothalamic AMPK (Figure 1), and leptin resistance may thus result from defects in AMPK-leptin signaling pathways as well as defects in the JAK2-STAT3 pathway. According to Gorobets (144), leptin resistance plays a role in body weight accumulation, and leptin resistance induced by APDs has been suggested to cause body weight gain in psychotic patients (145). For example, Piao et al. (146) demonstrate that risperidone activates extracellular-signal-regulated kinase (ERK) and blocks leptin-induced STAT3 phosphorylation over the course of treatment, enhancing both SOCS3 and SOCS6 mRNA expression. Interestingly, risperidone's impact on leptin and insulin pathways could not be explained by body fat accumulation (147). Other researchers have also examined leptin levels in patients receiving various APDs (mainly clozapine and olanzapine) (148). Several reports suggest that leptin levels in patients with psychosis might be higher than in healthy controls and that they may remain high during APDs therapy (149–151). Potvin et al. reported that olanzapine, clozapine, and quetiapine significantly increase leptin levels and show a correlation between leptin and BMI (149).

Margulska et al. (152) have demonstrated that changes in fasting serum levels of appetite-regulating peptides are not directly associated with the levels of clozapine in patients with schizophrenia. Furthermore, fasting serum levels of other peptides (CART, peptide YY, AgRP, deacylated ghrelin, and obestatin) do not exhibit meaningful correlations.

In summary, APDs have the potential to influence leptin levels directly or indirectly through the effect of obesity and subsequent leptin resistance. Thus, despite of the many studies focusing on this issue, the relationship between leptin levels and antipsychotics remains uncertain.

### Ghrelin and Adiponectin

The “hunger hormone” ghrelin is mainly produced by the stomach and stimulates appetite and appetite-related activities (153) (Figure 1). However, ghrelin has also been found to be distributed in other organs such as the pituitary, the lungs, the pancreas, the gall bladder, the esophagus, the colon, the liver, the spleen, the thyroid, the heart, and in the hypothalamic nuclei (ARC) (154–156). Ghrelin has two different forms, an acylated serine 3 form (ghrelin) and a des-acylated form (active form). The acylation of ghrelin is essential for binding to GHSR1a. Despite the appetite-promoting effect of ghrelin, it's mechanisms of action are not fully understood. An interesting study by Toshinai et al. demonstrates that the des-acylated form of ghrelin induces food intake independently of growth hormone secretagogue receptors (157). Other studies have shown that deficiencies of ghrelin have been linked to binge-eating and obesity (158, 159). Considering the molecular mechanism of appetite regulation, it has been reported that in the ARC and the hindbrain, ghrelin enhances food intake *via* activation of NPY and AgRP production (160, 161). However, both orexigenic (NPY/AgRP) and anorexigenic (POMC) neurons are shown to be modulated by ghrelin-expressing neurons (162). Moreover, ghrelin has been shown to activate AMPK in the hypothalamus to regulate food intake (163).

During treatment with some APDs, ghrelin has been reported to play a significant role in modulating appetite and energy homeostasis. Researchers have found that serum ghrelin levels in subjects treated with atypical APDs for at least 1 year are significantly higher than controls. An investigation shows a significant increase in total plasma ghrelin levels as well as active ghrelin after olanzapine administration after 6 months (164). Another study by the same group has shown that the level of active and total ghrelin in the risperidone group is significantly greater than that of healthy controls (165). In a preclinical study, it has been shown that olanzapine treatment elevates hypothalamic ghrelin receptor expression in rats (166, 167), supporting that ghrelin signaling plays a role in APD-induced obesity.

On the contrary, in a study on patients with schizophrenia, it was found that circulating ghrelin levels did not increase, but rather decreased, during treatment with risperidone or olanzapine, and there was also no significant difference between the effect of the two drugs (168). Similarly, in a meta-analysis by Goetz and Miller (169), it has been reported that patients with schizophrenia receiving olanzapine exhibit a significant drop in ghrelin levels. The reduction in ghrelin levels following olanzapine therapy may be due to obesity and excess energy intake. However, the drop in ghrelin levels may also be due to stress (170), which is common among psychotic patients. It is therefore questionable whether the drop in ghrelin level is the result of APDs treatment or stress.

Tagami et al. (171) used an electrical impedance-based receptor biosensor assay system (CellKeyTM) to study the effect of olanzapine on ghrelin-mediated GHSR signaling, reporting that olanzapine increased ghrelin-induced GHSR activity. Huang et al. reported that the 5HT2c receptor is dimerized with GHSR1a to inhibit orexigenic signaling, while 5HT2cR antagonists reduce dimerization and increase GHSR1a-induced food consumption (172). However, it is still necessary to establish the relationship between the 5HT2c receptor and GHSR, as well as and other receptor subtypes on appetite and obesity.

Adiponectin is an adipokine secreted by WAT and widely distributed in circulation. Adiponectin is known to promote fatty acid oxidation and insulin sensitivity in peripheral tissues by activating AMPK. A previous report (173) has shown that adiponectin increases AMPK activity in the ARC through its receptor AdipoR1, causing an increase in food intake. Moreover, mice deficient in adiponectin show reduced phosphorylation of AMPK in the ARC, resulting in a decrease in appetite, as well as higher energy expenditure.

Studies have shown that antipsychotic drugs can alter adiponectin levels. In a meta-analysis by Bartoli et al. (174), it has been found that people taking clozapine and olanzapine display lower adiponectin levels than those that are treated with risperidone. The lower adiponectin levels indicate reduced insulin signaling and are normally associated with metabolic disturbances. However, this meta-analysis does not correlate adiponectin levels with weight gain or other metabolic abnormalities. In a study by Lu et al. (175), APDs have been shown to impact adipokines levels and energy homeostasis, and olanzapine decreases the levels of adiponectin more effectively than clozapine. Other studies have found that adiponectin levels are also decreased by antipsychotics independent of adiposity and BMI (176). Thus, both APDs might influence adiponectin levels, but the mechanisms by which they do so have not been identified.

In conclusion, the dysregulated appetite and weight gain associated with atypical APDs seem to be associated with altered serum ghrelin and adiponectin levels or their signaling. Further research is needed to clarify the causality in this link and to explain the inconsistencies observed between several studies.

### Gut -Brain-Connection

**Figure 4:**
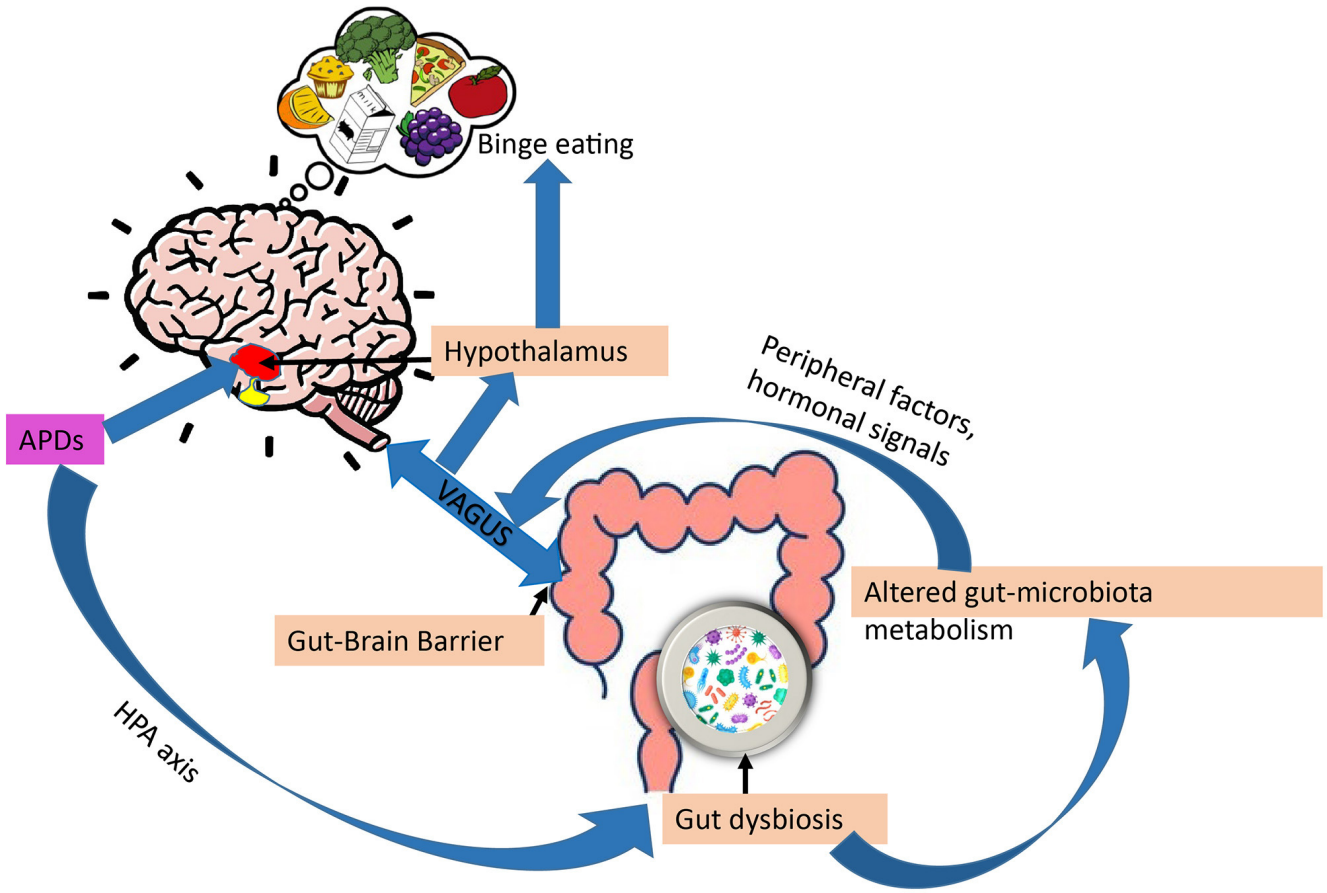
Gut-brain axis and regulation of appetite by APDs. Through impaired stress axis, antipsychotics (APDs) can influence gut microbiota composition, in turn, gut dysbiosis can alter gut-microbiota metabolism, changing the metabolites, leading to increased overeating and weight gain *via* peripheral (leptin, ghrelin) and vagal afferent signaling.

The possible role of the gut-brain axis in APD appetite regulation has been subject to attention during recent years (Figure 4). The brain is connected to the gut in several ways, including the vagus nerve, the hypothalamic-pituitary-adrenal axis, hormones, and metabolites, as well as through the immune system (177). Additionally, gut microbes have been shown to metabolize ingested nutrients to produce signaling molecules involved in the regulation of the stress axis, energy homeostasis, and obesity (178–180). Also, gut microbes regulate bile acid metabolism to produce a range of metabolites, including short-chain fatty acids (SCFAs), neurotransmitters, tiny protein molecules, and toxins (181). These metabolites may act directly as endocrine factors in addition to transmitting information to the hypothalamic circuitry through the vagal nerve. Both the VMH and ARC act together to sense peripheral metabolic and nutrient signals, thereby controlling appetite and glucose homeostasis (182).

APDs have been shown to alter gut microbiota compositions. For example, mice treated with olanzapine have altered gut microbiota compositions associated with obesity (183). According to Maier et al. (184), APDs can significantly reduce the level of *Akkermansia mucini* (human intestinal mucin degradation bacteria) in patients. An inverse relationship between the number of *Akkermansia mucini* and insulin resistance, as well as inflammation, has been demonstrated in mice. This supports the possibility that APDs may alter glucose metabolism directly *via* gut dysbiosis, which in turn can contribute to APD-induced obesity (34). Additionally, a study by Bahr et al. (185) has shown that risperidone increases body mass in children, reduces energy expenditure, and alters microbial composition. In another study by Yuan et al. (186) establishes that risperidone treatment increases body weight in first-episode schizophrenia patients and alters their gut microbiota.

We mentioned previously that gut microbiota metabolize food to generate signaling molecules involved in energy homeostasis. By altering the metabolites produced by dysbiosis of the gut, APDs have been shown to disrupt energy homeostasis. SCFAs, the main gut metabolites, have been shown to increase the ratio of AMP/ATP by activating AMPK (187–189). It has been observed that SCFA levels in feces are 20% higher in obese individuals than in lean individuals (190, 191). Therefore, increasing SCFA is related to obesity. A study has shown that olanzapine increases plasma SCFA levels in rats (192, 193). Moreover, antipsychotics have also been reported to change gut- microbiota metabolism through vitamin B6 (194). Thus, confirming APDs can alter gut-microbiota metabolism, thereby contributing to eating behavior and weight gain mediated by the gut-brain axis (195, 196).

Antipsychotics have been shown to impair the hypothalamic-pituitary axis (HPA) axis in psychotic disorders (178). On the other hand, several reports suggest that mental disorders and stress are also associated with gastrointestinal symptoms, and altered gut microbiota may thus be caused by the disease *per se*. and not by the pharmacological treatment (186, 197, 198). There are many studies demonstrating that the microbiome composition is less healthy in many treatment-naive patients with schizophrenia, bipolar disorder, and major depressive disorder (MDD) with psychotic features in comparison with healthy individuals (186, 199). Thus, this raises the question of whether gut-dysbiosis is mainly caused by APDs or the underlying psychotic disorder.

Overall, the alteration of gut microbiota composition by APDs may disrupt the gut-brain axis, which represents a potential mechanism of action in how these drugs induce appetite and disrupt energy homeostasis.

## Summary

Several antipsychotic drugs trigger weight gain, which is primarily due to an increase in food consumption. As discussed in this review, this effect is, at least in part, caused by the receptor binding profiles of these drugs and how they affect pathways in brain areas involved in appetite regulation. A high affinity for dopamine D2, serotonin 5HT2c, and histamine H1 receptors seems to be of great importance. Among antipsychotics with a high affinity for the 5HT2c receptor, clozapine and olanzapine cause the greatest weight gain and hyperphagia. Further, antagonistic effects on muscarinic and histaminergic receptors likely contribute to increased food consumption and metabolic dysfunction.

Considering the intercellular mechanisms involved, it has been shown that blocking H1 and 5HT2c receptors stimulate AMPK activity in the hypothalamus, which affects feeding through modulation of neuropeptide expression in ARC.

Other pathways that could be involved in the regulation of APD-induced dysregulated food intake include the endocannabinoid system and BDNF-mediated signaling. APDs seemingly do not have a direct effect on the cannabinoid system in regulating food intake. However, multiple neurotransmitter systems which might indirectly control CB1 receptors are shown to be affected by APDs. For example, olanzapine induces a reduction in CB1R density in both the hypothalamus and brain stem that stimulate the NPY and enhance inhibitory GABAergic input, thereby inhibiting the POMC and causing increased food intake. BDNF is a protein that may affect eating behavior, hormone release and activity, and energy homeostasis. The use of APDs has been shown to alter BDNF levels in the brain, cerebrospinal fluid (CSF), and serum in patients with schizophrenia, as reflected in animal studies. Nevertheless, to what degree BDNF activity may contribute to the obesity-related side effects of APDs is uncertain.

Finally, the enteric nervous system has been shown to have a complex relationship with gut microbiota and the central nervous system that can regulate food intake. Specifically, APDs have been found to affect the gut microbiota composition in such a way that it encourages overeating and weight gain *via* hormonal (leptin and ghrelin) and nutrient signaling *via* vagal afferents.

Unfortunately, there are currently no superior strategies available to prevent antipsychotic-induced hyperphagia or obesity. Consequently, a better understanding of the food intake controlling mechanisms that contribute to specific health impairments during APDs may assist in the creation of specific treatment strategies.

## Author Contributions

SM drafted the article which was critically reviewed by all authors during the development of the article. All authors listed have made a substantial, direct, and intellectual contribution to the work and approved it for publication.

## Funding

This work was funded by EuroNanoMed III (Grant No. EURONANOMED2019050/ENAMEP).

## Conflict of Interest

The authors declare that the research was conducted in the absence of any commercial or financial relationships that could be construed as a potential conflict of interest.

## Publisher's Note

All claims expressed in this article are solely those of the authors and do not necessarily represent those of their affiliated organizations, or those of the publisher, the editors and the reviewers. Any product that may be evaluated in this article, or claim that may be made by its manufacturer, is not guaranteed or endorsed by the publisher.
